# Monitoring Biofortification Program Performance and Potential for Impact: Indicators, Methods, and Learnings from the Commercialization of Biofortified Crops Program in Six Countries across Africa and Asia

**DOI:** 10.1016/j.cdnut.2024.104498

**Published:** 2024-10-29

**Authors:** Valerie M Friesen, Bho Mudyahoto, Annette M Nyangaresi, Ishank Gorla, Mduduzi NN Mbuya

**Affiliations:** 1 Global Alliance for Improved Nutrition, London, United Kingdom; 2 HarvestPlus, International Food Policy Research Institute, Kampala, Uganda; 3 Global Alliance for Improved Nutrition, Nairobi, Kenya; 4 Global Alliance for Improved Nutrition, New Delhi, India; 5 Global Alliance for Improved Nutrition, Washington, DC, United States

**Keywords:** biofortification, monitoring, program performance, indicators, Kenya, Tanzania, Nigeria, Pakistan, India, Bangladesh

## Abstract

**Background:**

Biofortification of staple crops is a food-based strategy to reduce the high global burden of micronutrient deficiencies. Monitoring program performance is essential to ensure biofortification programs have high potential for impact; however, few indicators and methods for doing so are publicly available.

**Objective:**

We documented the set of standardized indicators and methods used to monitor the Commercialization of Biofortified Crops (CBC) program and reviewed their strengths and limitations.

**Methods:**

Following the CBC program impact pathway, we identified and defined a set of indicators and corresponding methods. Country-level implementation teams contextualized and operationalized them to monitor 9 country-crop programs (i.e., high iron beans in Kenya and Tanzania, iron pearl millet in India, vitamin A maize in Nigeria and Tanzania, vitamin A cassava in Nigeria, zinc wheat in Pakistan and India, and zinc rice in Bangladesh) from 2020 to 2022.

**Results:**

Twenty indicators were defined across domains of seed supply, production, availability, awareness, capacity development, advocacy, and consumption of biofortified foods. Data collection methods included external and internal document review, farmer household surveys, rapid market assessments, and modeling. The strengths of these methods were that they were rapid to conduct, low cost, and simple to use. For some methods, the limitations were the potentially reduced accuracy of some results due to the use of external data sources or secondary data inputs and unavailability of data.

**Conclusions:**

The indicators and methods used in the CBC program are practical and cost effective for monitoring the implementation of biofortification programs because they generate the range of information necessary to understand how effectively a program is delivered and bolster plausibility arguments for attributing observed impacts to program activities. Further testing is needed to confirm their generalizability when applied to different contexts and paired with impact evaluations with the aim of producing publicly available global guidance documents.

## Introduction

The global burden of micronutrient deficiencies is staggeringly high, with an estimated half of preschool-aged children and two-thirds of nonpregnant women of reproductive age being deficient in at least one micronutrient [1]. Biofortification, i.e., the process of increasing the density of micronutrients in food crops through techniques such as conventional plant breeding, transgenic techniques, or agronomic practices, is a food-based strategy that may help alleviate this burden by increasing intakes of micronutrients that are commonly lacking in the diets of target populations [2]. The consumption of biofortified foods and food products has been shown to improve micronutrient intakes and status in controlled efficacy trials for several different crops and population groups [3] and in the few effectiveness studies that have been carried out to date [4,5].

Since biofortification began in the 1990s, its use has been increasing steadily around the world. As of 2024, >400 biofortified crop varieties of 12 staple food crops were released in 40 countries [6]. Biofortification programs typically include a variety of activities across crop-specific agricultural value chains. For example, agriculture research for varietal development, varietal release, agricultural input supply, farming, aggregation, processing, and retailing/public distribution. Together, these activities ultimately aim to increase consumption of essential micronutrients through biofortified staple foods in the target populations. As such, it is essential to monitor program performance (i.e., implementation progress and fidelity) across the entire value chain. Doing so can provide important information to understand whether programs are being implemented as designed, assess the extent to which they are achieving their objectives, and demonstrate plausible attribution of program activities to any impacts seen on micronutrient intakes or related nutrition and health outcomes.

For industrial food fortification programs, global guidance is publicly available on how to monitor program performance and impact across import, production, and commercial levels, and impact at household and individual levels [[7], [8], [9], [10]]. Furthermore, standardized indicators and data collection methods have been developed and widely implemented across countries [11,12]. However, for biofortification programs, similar global guidance and standardized indicators and data collection methods are not yet widely available. Most biofortification programs implemented to date have tracked a variety of project-level indicators, which are reported mainly in the gray literature (e.g., donor and institutional reports). For example, HarvestPlus, the leading organization in the development and promotion of biofortified crops, reports regularly on a number of seed supply-, production-, and household-level indicators. These include the number of varieties released, quantity of biofortified seed distributed, number of households reached with biofortified planting materials, number of farming households growing biofortified crops, and total estimated beneficiaries in farming households that consume biofortified foods [13,14]. However, the detailed definitions and data collection methods for these indicators are not publicly available. Conversely, a set of household-level coverage indicators was developed and pilot-tested based on similar indicators that are widely used in industrial fortification, i.e., consumption of the food, awareness of the biofortified food, availability of the biofortified food, consumption of the biofortified food (ever), and consumption of the biofortified food (current) [15]. However, these indicators have only been implemented in one province in Rwanda to date [16]. In 2022, Rodas-Moya et al. [17] recommended a set of 17 indicators (with corresponding data collection methods and tools) that cover different aspects of production, retail, and consumption of biofortified foods. These indicators were based on a review of existing biofortification programs; however, all but one are taken from program documentation that is not in the public domain.

The Commercialization of Biofortified Crops (CBC) program, led jointly by the Global Alliance for Improved Nutrition (GAIN) and HarvestPlus from 2019 to 2022, aimed to increase the production and consumption of biofortified foods by catalyzing commercial markets in 6 countries in Africa and Asia (i.e., Kenya, Tanzania, Nigeria, Pakistan, India, and Bangladesh) [18]. These 6 countries were selected because they had *1*) high levels of micronutrient deficiencies, *2*) GAIN and HarvestPlus presence, and *3*) at least one existing biofortified crop in production. During its inception phase in 2019, GAIN and HarvestPlus defined a set of standardized indicators and corresponding methods to track performance and estimate the potential impact of the CBC program. These indicators were then operationalized across all countries during the program implementation phase from 2020 to 2022. In this paper, we document the set of indicators and methods that were used to monitor the CBC program and review their strengths and limitations.

## Methods

### Defining indicators and corresponding data collection methods

In 2019, a generic theory of change for biofortification was developed by a collective of monitoring, evaluation, learning, and impact assessment specialists from HarvestPlus, GAIN, International Potato Center, Standing Panel on Impact Assessment, the International Center for Tropical Agriculture, and Wageningen University and Research [19]. Using this generic theory of change as a starting point, a technical support team (comprised of staff from GAIN and HarvestPlus with expertise in program design, monitoring, and evaluation) mapped out a detailed program impact pathway (PIP) for the CBC program during its inception phase [20]. To do this, several meetings were held during which the team interrogated and revised the generic theory of change. The aim was to ensure it accounted for all the nodes that would be relevant for the CBC program based on the planned activities and understanding of the program theory. Disagreements during this process were resolved through discussion among the team members. The technical support team then identified and mapped a series of indicators aligned to the nodes in the PIP. To do this, several meetings were held during which the team interrogated each node of the PIP. The aim was to determine what if any, indicators would be relevant to the program and its objectives and feasible in terms of data collection and analysis. During these meetings, the team reviewed existing indicators used by different organizations and specific indicators required by the donors and proposed new indicators. Disagreements during this process were resolved through discussion among the team members. Prior to use, the final PIP and list of indicators were then reviewed and approved by the senior management team for the CBC program (comprised of staff from both GAIN and HarvestPlus) to ensure alignment with program objectives and donor requirements.

The CBC PIP illustrates the causal pathways through which delivery of the CBC program would occur that would be essential for scaling up biofortification through commercial markets. Specifically, it outlines 4 main pathways through which people could consume biofortified foods, i.e., purchased by consumers from markets, given to consumers in informal settings (e.g., friends and family), given to consumers in formal settings (e.g., institutional food distribution programs such as school feeding programs or public distribution systems), and allocated for home consumption from onfarm production (by farming households), of which the first 3 were considered commercial under the CBC program. The selected indicators can enable the capture of essential information on the various domains that the program activities aim to influence. Specifically, indicators were selected that could track program performance (i.e., implementation progress and fidelity) by considering where the program activities fall in the PIP and the relevant output-, outcome-, and impact-level results, as well as other internal or donor specific information needs. For each indicator that was identified, the technical support team developed a template performance indicator reference sheet (PIRS) that included the precise definition, method of calculation, appropriate and feasible data collection methods, data sources, and reporting frequencies [21]. In addition, the technical support team developed templates for other accompanying tools, including a country-level monitoring and evaluation plan, country- and global-level results frameworks, and data collection forms.

### Contextualizing and operationalizing the indicators and data collection methods

The CBC program included 9 country-crop combinations, i.e., high iron beans in Kenya and Tanzania, iron pearl millet in India, vitamin A maize in Nigeria and Tanzania, vitamin A cassava in Nigeria, zinc wheat in Pakistan and India, and zinc rice in Bangladesh. All the country-crop combinations focused on the commercial market pathway except for high iron beans and vitamin A maize in Tanzania, which focused predominantly on the institutional pathway through school feeding programs. Country-level implementation teams (comprised of a project manager from both GAIN and HarvestPlus and, in some cases, a monitoring and evaluation specialist in each of the 6 countries) contextualized the indicators and data collection methods for each of the 9 country-crop combinations. To do this, implementation teams reviewed the PIP and list of standard indicators and selected the relevant indicators that aligned with their commercialization strategy. Although most indicators were tracked for all country-crops, if a country-crop strategy did not include activities that focused on the institutional commercialization pathway or biofortification policy development, then the related indicators were removed. Disagreements during this process were resolved through discussion between the implementation team and technical support team members. Then, based on the templates developed by the technical support team, the implementation teams developed country-crop specific monitoring and evaluation plans that included a PIRS for each indicator and overall results framework as well as other tools (e.g., data collection forms). The implementation teams contextualized these tools to the specific crop and added further details such as those responsible for data collection, frequency of data collection and reporting, targets, etc.

Throughout the 3 years of implementation of the CBC program (2020–2022), the implementation teams operationalized these standardized indicators and data collection methods. Specifically, the implementation teams and technical support team collected and analyzed data in accordance with their country-crop specific monitoring and evaluation plan and PIRS. For indicators that used document review as the data collection method, the implementation teams collected and analyzed the data. For indicators that used farmer household surveys as the data collection method, the technical support team members from HarvestPlus led the design, data collection, and analysis, and the implementation teams reviewed and provided input on the results. For indicators that used rapid market assessments, a subcontracted third party (i.e., Wellspring) led the design, data collection, and analysis, and the implementation teams and technical support team reviewed and provided input on the design and results. For indicators that used modeling, the technical support team led the data collection and analysis, and the implementation teams reviewed and provided input on the results. Farmer household surveys were submitted to the International Food Policy Research Institute Institutional Review Board for approvals but were deemed internal quality improvement activities, not research with human subjects, and thus were exempt. All other methods were not submitted for ethical approval because they did not meet the definition of research with human subjects. Additionally, the implementation teams collated the results, which formed the basis for monthly and quarterly program monitoring and annual donor reporting. The technical support team subsequently collated results annually across the 9 country-crop combinations in a global results framework that was used for donor reporting. Results were used to track program performance, identify implementation bottlenecks, and inform corrective actions where needed. As part of the end of program reporting in 2022, the technical support team documented the strengths and limitations of the indicators and data collection methods based on feedback from the implementation teams and the technical support team members.

## Results

**FIGURE 1:**
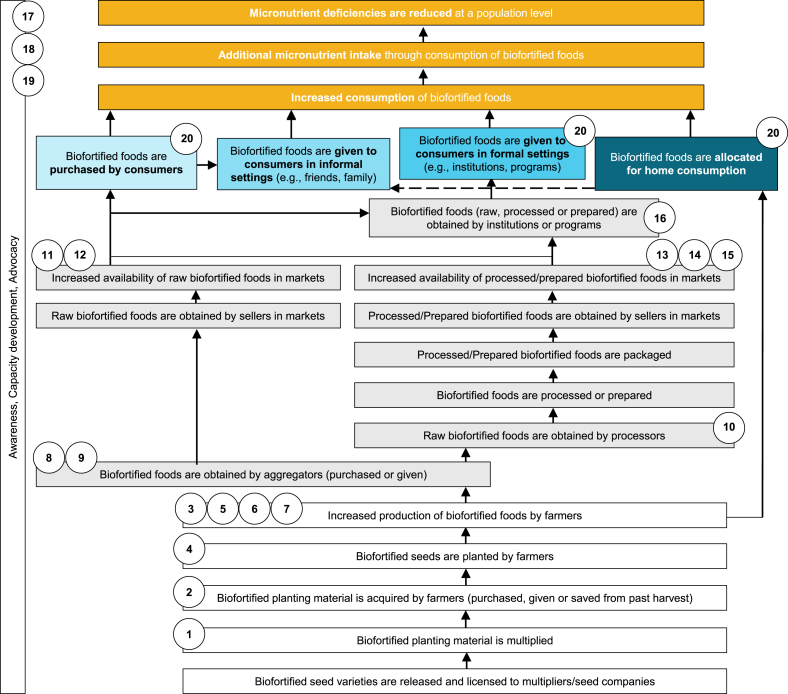
Indicators for monitoring the Commercialization of Biofortified Crops aligned to its program impact pathway. Refer to Table 1 for detailed descriptions of indicators by number shown here. Reproduced from Friesen et al. [20] under the Creative Commons Attribution-Non-Commercial-Share Alike 4.0 IGO license (CC BY-NC-SA 4.0 IGO; https://creativecommons.org/licenses/by-nc-sa/4.0/).

Twenty indicators were defined along with their corresponding data collection methods and additional details (e.g., data source, method of calculation, and reporting frequency). These indicators were aligned to 13 of the 22 nodes in the PIP and cut across the different domains of seed supply, production, availability, awareness, capacity development, advocacy, and consumption of biofortified foods (Figure 1 and Table 1). Indicators for seed supply included the quantity of biofortified seed/planting material available and the number of farmers that acquire biofortified seed/planting material. These indicators were based on external document review and data collected from farmer household surveys. Indicators for production included the quantity of biofortified harvested food, the number of farmers that grow and sell biofortified foods, and the number of farmers that report increased production and income from the sale of biofortified foods. These indicators were based on data collected from farmer household surveys. Indicators for availability included the quantity of biofortified food obtained by aggregators and the volumes of raw and prepared or processed biofortified foods and food products in the market and/or distributed through formal institutions based on data collected from external document reviews and rapid market assessments. Additionally, availability indicators included the number of different value chain actors (i.e., aggregators, processors, and retailers) procuring and/or selling biofortified foods and food products based on external document reviews. Indicators for awareness and capacity development included the number of value chain actors across the entire value chain that are aware of biofortified foods and their benefits and those who received capacity development support (e.g., technical and/or financial) based on data collected from internal document reviews. Indicators for advocacy included the number of policies, strategies, plans, or legislation documents that mention biofortified foods in the country at any level (e.g., local, regional, or national) based on data collected from external document reviews. Finally, the indicator for consumption was the number of people who consume biofortified foods. This indicator was modeled based on results from other indicators of production and availability (e.g., annual supply volume of biofortified food or number of farmers growing biofortified crops) and, for commercial pathway estimates, additional secondary data inputs (i.e., on losses due to processing methods and annual consumption of the food).

**TABLE 1:** Indicators used for monitoring the Commercialization of Biofortified Crops program and their data collection methods, data sources, methods of calculation, disaggregations, and reporting frequencies^1^.

Expected result	Indicator (unit)	Data collection method	Data source	Method of calculation	Disaggregation	Reporting frequency
1. Supply of biofortified seed increased	1. Quantity of seed/planting material available that is biofortified (MT or number of VAC stems)	External document review	Sales/distribution records from seed producers/multipliers	Count and sum all seed quantities of biofortified crop varieties sold/distributed in the target geography.	Crop; Country	Annually
	2. Number of farmers that acquire biofortified seed/planting material (number)	Farmer household survey	Farmer household survey report	Take the quantity of seed/planting material available that is biofortified (indicator #1) and divide by the common seed pack size of biofortified varieties reported to be purchased by farmers.	Crop; Country; Sex of farmer	Annually
2. Production of biofortified foods increased	3. Quantity of harvested food that is biofortified (MT)	Farmer household survey	Farmer household survey report	First, count the number of farmers that reported growing the biofortified varieties of the crop at the end of the reporting period. Second, estimate the average yield attained by farmers for each crop. Third, estimate the average area planted by farmers for each crop. Fourth, estimate the total quantity of food produced/harvested that is biofortified by multiplying the 3 parameters above.	Food; Country	Annually
	4. Number of farmers that grow biofortified foods (number)	Farmer household survey	Farmer household survey report	Take the number of farmers that acquire biofortified seed/planting material (indicator #2) and adjust it for diffusion and disadoption rates established in the survey.	Crop; Country; Sex of farmer	Annually
	5. Number of farmers that report increased production of biofortified foods (number)	Farmer household survey	Farmer household survey report	Count and sum all the farmers growing biofortified crops that reported increased quantity of harvested biofortified crop compared to the previous reporting period. To estimate this indicator as a proportion, divide the number calculated above by the total number of all farmers that grow the biofortified crop in the reporting period and multiply by 100.	Crop; Country; Sex of farmer	Years 2 and 3
	6. Number of farmers that sell harvested biofortified foods (number)	Farmer household survey	Farmer household survey report	Count and sum all the farmers growing biofortified foods that sell it to VCAs. To estimate this indicator as a proportion: divide the number calculated above by the total number of all farmers that grow the biofortified crop in the reporting period and multiply by 100.	Crop; Country; Sex of farmer	Years 2 and 3
	7. Number of farmers that report increased income from sale of biofortified foods (number)	Farmer household survey	Farmer household survey report	Count and sum all the farmers growing biofortified crops that reported increased income from sale of harvested biofortified foods compared to the previous reporting period. To estimate this indicator as a proportion, divide the number calculated above by the total number of all farmers that grow the biofortified crop in the reporting period and multiply by 100.	Crop; Country; Sex of farmer	Years 2 and 3
3. Availability of raw biofortified foods and food products increased	8. Quantity of biofortified food obtained by aggregators (MT)	External document review	VCA records/databases from implementing partners and (where applicable) industry associations	Sum all quantities of biofortified food reported to be obtained by individual aggregators.	Food; Country	Annually
	9. Number of aggregators that procure biofortified foods (number)	External document review	VCA records/databases from implementing partners and (where applicable) industry associations	Count and add any new aggregators to the total of the previous reporting period.	Food; Country	Annually
	10. Number of processors that procure biofortified foods (number)	External document review	VCA records/databases from implementing partners and (where applicable) industry associations	Count and add any new processors to the total of the previous reporting period.	Food; Country	Annually
	11. Proportion of raw biofortified food volume available in the market (%)	Rapid market assessment	Rapid market assessment report	Count the number of retailers selling raw biofortified foods in the market. Then, estimate and sum the quantity available for each raw product at each retailer. Then, divide by the estimated total volume of food available in the market.	Food; Country	Year 3
	12. Number of retailers selling raw biofortified foods (number)	Rapid market assessment	Rapid market assessment report	Count the number of retailers selling raw biofortified foods in the market.	Food; Country	Year 3
	13. Number of prepared or processed food products available that contain biofortified food in the market (number)	Rapid market assessment	Rapid market assessment report	Count the number of prepared or processed food products available that contain biofortified food in the market.	Food; Country	Year 3
	14. Proportion of prepared or processed food volume available in the market (%)	Rapid market assessment	Rapid market assessment report	Count the number of retailers selling prepared or processed biofortified food products in the market. Then, estimate and sum the quantity available for each raw product at each retailer. Then, divide by the estimated total volume of food available in the market.	Food; Country	Year 3
	15. Number of retailers selling food products that contain biofortified food (number)	Rapid market assessment	Rapid market assessment report	Count the number of retailers selling food products that contain biofortified food in the market.	Food; Country	Year 3
	16. Quantity of biofortified food distributed through formal institutions/programs (in any form, i.e., raw or prepared/processed) (MT)	External document review	Food distribution records from institutions involved in biofortified food distribution schemes	Count and sum the quantities of biofortified food used in food distribution schemes by institutions/programs during the reporting period.	Food; Country	Annually
4. Awareness of biofortification among value chain actors improved	17. Number of VCAs that are aware of biofortified foods and their benefits (number)	Internal document review	Internal attendance records from training/awareness events	Count and add any new VCAs to the total of the previous reporting period.	Food; Country	Annually
5. Biofortification integrated into policies and legal frameworks	18. Number of policies/strategies/plans/legislation documents that mention biofortified foods at any level (e.g., local, regional, national) (number)	External document review	Policy records from relevant authorities	Count and sum all documents of the same category per policy jurisdiction (e.g., local, regional, national). Record only new ones in each category during each reporting period.	Food; Country	Annually
Crosscutting – contributes to expected results 1, 2, and 3	19. Number of VCAs that received capacity development support (e.g., technical, financial) (number)	Internal document review	Internal training and financial records	Count all VCAs that received capacity development support during the reporting period. Record an institution that received equipment as one VCA.	Food; Country; Capacity development type	Annually
6. Consumption of biofortified foods increased	20. Number of people who consume biofortified foods (number)	Modeling	Results from other indicators and secondary data	Model based on production and availability data for each of the consumption pathways and then sum to get the total number of consumers, as follows: 1. For market pathway: annual supply volume of biofortified food in a country (from indicator #3) multiplied by the % of production sold in the market (from farmer household survey) multiplied by the milling/shelling ratio to account for losses due to processing methods (from secondary data) divided by the annual per capita food consumption [from secondary data, i.e., most recent food balance sheets [29]]. 2. For formal institution/program pathway: annual supply volume of biofortified food (from indicator #16) divided by the annual per capita food consumption [from secondary data, i.e., most recent food balance sheets [29]]. 3. For farmer home consumption pathway: number of farmers growing biofortified crops (from indicator #4) multiplied by the average number of household members in rural farming households (from farmer household survey).	Food; Country; Consumption pathway	Years 2 and 3

Abbreviations: MT, metric tons; VAC, vitamin A cassava; VCA, value chain actor (e.g., seed producers, farmers, aggregators, processors, retailers, and consumers)

1 Adapted from GAIN & HarvestPlus (21) based on what was actually done during program implementation.

The methods used to collect data to inform the 20 CBC program indicators were classified into 5 categories, i.e., external document review (e.g., sales/distribution records from seed producers/multipliers), internal document review (e.g., internal training records), farmer household surveys, rapid market assessments, and modeling, which each had different strengths and limitations (Table 2). Overall, the main strengths of the data collection methods were that they were generally rapid to conduct, had minimal costs, and were simple to use. The main limitations were the potentially limited accuracy of some results due to the use of external data sources (e.g., those used in external document reviews) or secondary data inputs (e.g., those used in modeling) and unavailability of data in some cases (e.g., in some countries for rapid market assessments).

**TABLE 2:** Strengths and limitations of the data collection methods used to inform the Commercialization of Biofortified Crops program monitoring indicators.

Data collection method	Strengths	Limitations
External document review^1^	- No cost to implement - Rapid to conduct - Limited technical expertise required to collect and analyze data	- Data used were not directly collected by program staff
Internal document review^2^	- No cost to implement - Rapid to conduct - Limited technical expertise required to collect and analyze data - Data were readily available from program implementation staff	- Accuracy of the records collected varied across program staff - The same data were sometimes collected in different ways by program staff
Farmer household surveys^3^	- Less expensive to implement (versus a traditional household survey) - Rapid to conduct (as these were phone-based surveys) - Annual implementation provided a good basis for tracking changes over time - Limited technical expertise required to analyze data	- Required resources (i.e., cost, time, and staff) as this was primary data collection - The surveys were designed to be representative of the areas in which they were carried out but did not cover all geographies where the program was implemented
Rapid market assessments^4^	- Can be easily replicated to generate updated estimates over time - Moving forward, the resources and technical expertise required to collect and analyze data should be reduced	- Required resources (i.e., cost, time, staff, and third-party service provider) as this was primary data collection - In some countries, it was difficult to obtain the necessary data to map the volume of biofortified food throughout the value chain (due to, e.g., limited awareness, invisible traits, mixing, or lack of segregation); therefore, the confidence in the estimates is lower in countries where data were limited - Only conducted once at the end of project as time was needed to develop the method
Modelling^5^	- No cost to implement - Data inputs were readily available as they came from other indicators and secondary data - Can be easily replicated to generate updated estimates over time	- Indirect measure of consumption - Technical expertise required to analyze data - Secondary data inputs used (e.g., annual per capita food consumption) may not be representative of all populations where the program was implemented - Did not quantify amounts of biofortified foods consumed, additional micronutrient intakes from biofortified foods, or changes in micronutrient status following consumption of biofortified foods, which are needed to truly understand impact on micronutrient intakes and status indicators

1 For indicators #1 Quantity of seed/planting material available that is biofortified, #8, Quantity of biofortified food obtained by aggregators, #9 Number of aggregators that procure biofortified foods, #10 Number of processors that procure biofortified foods, #16 Quantity of biofortified food distributed through formal institutions/programs (in any form, i.e., raw or prepared/processed), and #18 Number of policies/strategies/plans/legislation documents that mention biofortified foods at any level (e.g., local, regional, national).

2 For indicators #17 Number of value chain actors (e.g., seed producers, farmers, aggregators, processors, retailers, and consumers) that are aware of biofortified foods and their benefits, and #19 Number of value chain actors that received capacity development support, (e.g., technical, financial).

3 For indicators #2 Number of farmers that acquire biofortified seed/planting material, #3 Quantity of harvested food that is biofortified, #4 Number of farmers that grow biofortified foods, #5 Number of farmers that report increased production of biofortified foods, #6 Number of farmers that sell harvested biofortified foods, and #7 Number of farmers that report increased income from sale of biofortified foods.

4 For indicators # 11 Quantity of raw biofortified food volume available in the market, #12 Number of retailers selling raw biofortified foods, #13 Number of prepared or processed food products available that contain a biofortified food in the market, #14 Quantity of prepared or processed food volume available in the market, and #15 Number of retailers selling food products that contain a biofortified food.

5 For indicator #20 Number of people who consume biofortified foods.

Select results from this set of indicators were used to demonstrate the performance and potential for impact of the CBC program at both individual country and global levels and for institutional and donor reporting (Table 3). For example, throughout the program implementation period from 2020 to 2022, increases in the quantity of biofortified seeds, quantity of harvested food that was biofortified, and number of farmers who grew biofortified foods were observed across the geographies targeted by the program. These findings demonstrated that the program activities that were being implemented and generated the expected results of increasing seed supply and production of biofortified foods. Thirteen new policies, strategies, plans, or legislation documents that mentioned biofortification were produced in total in 4 of the 6 countries (i.e., Nigeria, Bangladesh, Tanzania, and Pakistan) during the 3 years of implementation. These findings demonstrated the success of the program’s advocacy efforts. Finally, the number of people who consumed biofortified foods increased from 2021 to 2022 (this indicator was not assessed in 2020 as the program activities were only starting up then). As the highest impact-level indicator assessed, these findings demonstrated the increasing potential for impact of the program to increase micronutrient intakes.

**TABLE 3:** Consolidated global results for select indicators monitored in the Commercialization of Biofortified Crops (CBC) program over the 3 y of implementation^1^.

Indicator	2020	2021	2022
Expected result 1: Supply of biofortified seed increased			
Quantity of seed/planting material available that is biofortified (MT or VAC stems)	2859 MT of seeds and 301,000 VAC stem bundles	16,550 MT of seeds and 596,094 VAC stem bundles	61,197 MT of seeds and 1,711,942 VAC stem bundles
Expected result 2: Production of biofortified foods increased			
Quantity of harvested food that is biofortified (MT)	172,589	21,734,394	27,159,637
Number of farmers that grow biofortified foods	368,290	8,292,946	8,318,552
Expected result 3: Availability of raw biofortified foods and food products increased			
Proportion of raw biofortified food volume available in the market (%)^2^	Not assessed	Not assessed	5 (0–15)
Number of prepared or processed food products available that contain biofortified food in the market	Not assessed	Not assessed	77
Quantity of biofortified food distributed through formal institutions/programs (in any form, i.e., raw or prepared/processed)^3^ (MT)	0	173	2266
Expected result 4: Awareness of biofortification among VCAs improved			
Number of VCAs that are aware of biofortified foods and their benefits	1,240,067	45,513,387	36,269,915
Expected results 5: Biofortification integrated into policies and legal frameworks			
Number of policies, strategies, plans, or legislation documents that mention biofortified foods at any level (e.g., local, regional, national)^4^	5	4	4
Crosscutting – contributes to expected results 1, 2, and 3			
Number of VCAs that received capacity development support (e.g., technical, financial)	1811	95,648	438,737
Expected result 6: Consumption of biofortified foods increased			
Number of people who consume biofortified foods	Not assessed	133,925,212	186,195,309

Abbreviations: MT, metric tons; VAC, vitamin A cassava; VCAs, value chain actors (e.g., seed producers, farmers, aggregators, processors, retailers, and consumers).

1 Results shown are the sum of the individual results for all 9 country-crop combinations in the CBC program (i.e., high iron beans in Kenya and Tanzania, iron pearl millet in India, vitamin A maize in Nigeria and Tanzania, vitamin A cassava in Nigeria, zinc wheat in Pakistan and India, and zinc rice in Bangladesh) except where indicated.

2 Results shown are the mean (range) of the individual results for all 9 country-crop combinations in the CBC program.

3 Results shown are the sum of the individual results for the 2 country-crop combinations that had commercialization strategies predominately focused on the formal institutional pathway (i.e., vitamin A maize and high iron beans in Tanzania, which focused on school feeding programs).

4 Of the 13 total policies, strategies, plans, or legislation documents, there were 6 in Nigeria, 3 in Bangladesh, 3 in Tanzania, and 1 in Pakistan.

## Discussion

As part of the CBC program, we defined and operationalized a set of standardized indicators and corresponding data collection methods to monitor program performance and estimate potential for impact (i.e., the number of people consuming biofortified foods) across 6 countries for 9 country-crop combinations (i.e., high iron beans in Kenya and Tanzania, iron pearl millet in India, vitamin A maize in Nigeria and Tanzania, vitamin A cassava in Nigeria, zinc wheat in Pakistan and India, and zinc rice in Bangladesh). These indicators and methods are practical and cost-effective for program planners, program implementers, and technical partners to track performance and potential for impact of biofortification programs across time and space. This is because they demonstrate the range of information necessary to understand the extent to which a program is being effectively delivered (across the entire PIP) and strengthen plausibility arguments for attributing observed impacts on nutrition and health outcomes to program activities. Furthermore, they generate information that can be used by governments and donors to track value for money and collate results across different investments.

The CBC program indicators built on indicators that were previously defined and used to monitor and evaluate biofortification and industrial fortification programs, and also included several new ones, as follows.

The seed supply- and production-level indicators selected aligned with or were slightly modified from those that had previously been used in biofortification programs by HarvestPlus but for which detailed data collection methods and calculations were not publicly available [13,14] and also included some new ones. Specifically, the number of farmers growing biofortified crops previously was retained. The quantity of biofortified seed distributed and total beneficiaries reached with biofortified planting materials were modified to the quantity of biofortified seed available and the number of farmers that acquire biofortified seed. An additional indicator was included for the quantity of biofortified harvested food. These changes were made to ensure alignment with the specific aspects articulated in the CBC PIP. Furthermore, additional indicators on the number of farmers that reported increased production of biofortified foods and increased income from sales of biofortified foods were also included because they were priorities for one of the donors. Together, this set of indicators was useful to identify program implementation bottlenecks at the critical production level to track the progress of biofortified foods through the value chain from farms to commercial markets and to understand the impact on farmers’ livelihoods.

The availability-level indicators (i.e., food volume at aggregation and market levels and number of aggregators/processors/retailers and biofortified food products at market level) are new and have not been previously published or reported on in biofortification programs to our knowledge. These were developed by building on similar indicators defined to assess industrial fortification programs [11,22] and are essential to understanding the flow of biofortified foods post farm gate and provide critical information to identify program implementation bottlenecks and opportunities for commercialization.

The consumption-level indicator (i.e., the number of people consuming biofortified foods) aligned with indicators previously used by HarvestPlus and others but went further to ensure it could be disaggregated by the main pathways through which people consume biofortified foods [i.e., purchased from markets, received from formal institutions/programs, and allocated for home consumption from onfarm production (by farming households)]. This distinction was important for the CBC program because it focused on catalyzing commercial pathways. HarvestPlus regularly reports on the total beneficiaries reached with biofortified foods (in farming households) [13], and the set of household coverage indicators tested in Rwanda includes the number of households consuming biofortified food (current) (without any disaggregation by pathway) [15]. Higher impact-level indicators beyond consumption were not included in the CBC program. This was due to the short duration of the program implementation (3 years) and limited resources available to conduct household-level surveys to directly measure this and/or other impact indicators. That said, once a biofortification program has reached a stage of maturity at which there is a high volume of biofortified foods in the market that would warrant conducting a household-level survey, additional indicators that are aligned to those used in industrial fortification (e.g., amount of biofortified food consumed, micronutrient intakes from biofortified foods, and micronutrient adequacy or status at the individual level) [7,11] could be measured.

There were 3 key learnings generated from the different data collection methods used to inform the CBC program indicators that have implications for their future use. First, when prioritizing methods that are rapid, low cost, and easy to implement, there will often be tradeoffs in terms of accuracy. For example, the farmer household surveys were annual cross-sectional telephone-based surveys that were conducted in a randomly determined subsample of all farmers in geographies that represented program implementation areas, and results were extrapolated to the national level. Although this design made for more rapid and less expensive data collection, the results may not have been representative of farmers in all areas of the country. For a small-scale program, this is likely fine; however, in a national-level program aimed at scaling biofortified foods in commercial markets, making assumptions at a national level based on a subsample of the population may result in over- or underestimation of results.

Second, although the rapid market assessment methods have the potential to generate critical data to understand how biofortified foods flow post farm gate, further refinement and testing are needed because there were some challenges in collecting data to inform these indicators on biofortified food volume/products. The main challenge was obtaining data for crops that flowed through informal markets; therefore, confidence in the estimates may be limited in some countries. Informal markets were largely the case for many of the crops included in the CBC program. For example, in Nigeria, >95% of vitamin A cassava tubers produced are processed into fufu and gari by microscale processors and sold unpackaged and unbranded through local open markets, kiosks, street stores, and/or directly to local restaurants [23]. Additional issues around aggregation and segregation were identified. For example, in Pakistan, there was limited segregation between zinc wheat and conventional wheat varieties at the aggregation and processing stages. This was reportedly due to a lack of market demand and existing formal government policy or mechanism for segregation. These challenges make it difficult to identify methods that enable accurate estimation of indicators related to the availability of biofortified foods and food products (i.e., indicators #11 to #15). This is especially true for a program such as biofortification, which is just beginning to focus on commercial markets (as opposed to its initial focus in previous decades on smallholder farmers). That said, with further testing and refinement, the rapid market assessment method could be a game changer in tracking the scale-up of biofortification through commercial markets. The market share model developed under the CBC by Wellspring (i.e., the third party that was engaged to support defining the methods and carrying out the data collection and analysis) was designed so that it can be easily replicated and used to track changes over time. Moving forward, other market-level data collection efforts could also be explored to help inform this indicator (e.g., through market research companies that regularly track price and volume of diverse food and other products). This has been explored for industrial fortification in recent years, e.g., market volumes of oil industrially fortified with vitamin A were assessed in Bangladesh to understand how well the mandatory fortification program was functioning [22].

Third, the choice of methods used to assess impact-level indicators must be aligned to the program objectives and resources available to ensure they are feasible yet rigorous enough to still provide meaningful results for the program. Under the CBC program, the highest impact-level indicator, i.e., number of people consuming biofortified foods, was modeled using other production-level indicator values (e.g., annual supply volume of biofortified food or number of farmers growing biofortified crops) and, for commercial pathway estimates, additional secondary data inputs (i.e., on losses due to processing and annual consumption of the food). This highlighted a major challenge with using data from so far down the PIP to inform impact-level indicators with limited knowledge of what happens at the steps in between. Availability-level data from the rapid market assessments were planned to be incorporated into the calculation of the consumption indicator in the third year of the program. This was intended to improve accuracy by using data inputs that were closer to the consumer in the PIP and, thus, more likely to represent true consumption. However, this was not ultimately done due primarily to challenges with unavailable data in some cases (as described above). Although a household-level survey would likely be the most accurate method to measure consumption, it was not prioritized in the CBC program, given the short duration of the program and the long PIP that the program activities aimed to tackle. As such, modeling consumption based on availability data made the most sense. Moving forward, the decision of what data inputs to use when estimating the number of people consuming biofortified foods should be dependent on the maturity, duration, and objectives of the program as well as resources available to decide when to measure consumption more directly or go further to assess changes in micronutrient intake or status through consumption of biofortified foods. Similar to the global guidance on when to conduct an evaluation of a large-scale food fortification program [7], there is a need to ensure that the biofortification program has met a certain level of operational performance to warrant investment in such measurement.

In addition to routine program monitoring, there are other complementary research activities that can be employed during the implementation of a biofortification program to understand different aspects of performance and potential for impact. For example, implementation research can be conducted, which aims to go beyond how well a program is performing to understand the enablers and barriers to program delivery within and across the different domains that affect implementation and to test the validity of the assumptions in the PIP [24]. It has been used in industrial food fortification programs, e.g., to understand enabling factors related to double-fortified salt adherence in India [25]. Given the short duration of the CBC program and the limited budget available, implementation research was only conducted in India for the zinc wheat and iron pearl millet programs. Specifically, baseline and ongoing data collection were conducted across all program domains, and results were used to examine the program theory, understand issues of fidelity of implementation, and identify course corrective actions over the last 1.5 y of the program. For example, the invisible traits of the 2 biofortified foods were identified as key challenges to traceability and aggregation of the grains. As a result, implementation teams adjusted their activities to better streamline the aggregation process, which led to some incremental improvements by the end of the program. Given the nascency of efforts to commercialize biofortified foods, implementation research can play a critical role in closing the gap between understanding how interventions are intended to work and realizing actual impacts in programs implemented at scale, a priority that has been recently highlighted as essential in nutrition interventions more broadly [24,26].

The major strengths of this set of indicators and methods were that it enabled the standardization of results across countries and crops, collation of results in a global results framework for reporting, and opportunities for cross-country learning in the context of a large, diverse, multicountry program. At the same time, there were some limitations. First, although the 20 indicators identified were aligned to the PIP, none of them specifically related to gender considerations (apart from the possibility of disaggregating those related to value chain actors by sex) or environmental impacts. There is a growing body of evidence that shows that improving the position of women in terms of control of household resources, bargaining power within the household, and agricultural productivity leads to greater impacts on health and nutrition outcomes [27]. Additionally, biofortification programs are often promoted as a mitigation strategy to tackle the negative effects of climate change, which have been shown to reduce the yield and micronutrient contents of foods [28]. Future work should, therefore, assess the extent to which these gender- and environment-related factors have been influenced and assessed in other biofortification programs and/or explore testing of relevant and sensitive indicators linked to these factors to better understand how they may influence biofortification program performance and potential for impact. Second, although the indicators and methods were designed to be applicable in any setting and tested across 9 country-crop combinations in Africa and Asia that focused on commercialization efforts, these countries did not include the full range of geographies where biofortification programs are being implemented, nor all the different biofortified crops that have been released to date. Further testing is recommended to confirm the generalizability of these indicators when applied to different geographies and crops as well as programs with different aims (e.g., beyond commercialization).

The indicators and methods developed and operationalized under the CBC program are practical and cost-effective tools for monitoring biofortification programs because they demonstrate the range of critical results along the PIP needed to understand program delivery and bolster plausibility arguments for attributing observed impacts to program activities. Moving forward, further testing and refinement are needed to confirm their generalizability when applied to different contexts and paired with impact evaluations with the aim of producing publicly available guidance documents that can be implemented in biofortification programs globally. This is essential given the current stage of biofortification, which is a time marked by increasing efforts to expand and scale-up biofortification around the world. The availability and use of a harmonized set of indicators and methods for monitoring would thus enable the tracking of progress, scale-up, and impact of biofortification programs over time.

## Author contributions

The authors' responsibilities were as follows – VMF, BM, MNNM: designed research; VMF, BM, AMN, IG, MNNM: conducted research; VMF: analyzed the data and wrote the paper; BM, AMN, IG, MNNM: critically reviewed the manuscript; VMF: had primary responsibility for final content and all authors: read and approved the final manuscript.

## Funding

Supported by German Federal Ministry of Economic Cooperation and Development (BMZ) and the Netherlands Ministry of Foreign Affairs. Funders had no role in the study design, collection, analysis, interpretation of data, writing of the manuscript, or the decision to submit for publication.

## Data sharing

Data described in the manuscript will be made available upon request to datasharing@gainhealth.org.

## Conflict of interest

The authors report no conflicts of interest.
